# Supplementary material for: Establishing a framework towards monitoring HCV microelimination among men who have sex with men living with HIV in Germany: A modeling analysis
Source: PLoS One. 2022 May 12;17(5):e0267853. doi: 10.1371/journal.pone.0267853 (PMC9098082; doi:10.1371/journal.pone.0267853)
Supplement: S1 Table — HCV: hepatitis C virus; MSM: men who have sex with men; SVR: sustained virological response; IFN/RBV: Interferon/Ribavirin; UK: United Kingdom. (DOCX) [file pone.0267853.s001.docx]

**S1 Table. Prior and posterior ranges for parameters which were varied for model fitting.** HCV: hepatitis C virus; HIV: human immunodeficiency virus; MSM: men who have sex with men; SVR: sustained virological response; ART: antiretroviral therapy; IFN/RBV: Interferon/Ribavirin; UK: United Kingdom; I: interval; CI: confidence interval; IQR: interquartile range; AOR: adjusted odds ratio.

| **Parameters varied for fitting** | **Prior range, distribution/ Posterior mean [95% Interval]** | **Comments** |
| --- | --- | --- |
| **Proportion of HIV-positive who spontaneously clear acute HCV infection** | Prior: 9-15%, uniform  Posterior: 11.6% [95% I 9.3-14.3%] | [1]  [2]  [3] |
| **Proportion of those infected who do not spontaneously clear initiated onto treatment within 6 months of diagnosis** | Prior range: 75-85%, uniform  Posterior: 76.9% [95% I 75.2-79.8%] | High treatment rates seen across Germany (55-83% of acute infections treated each year during 2007-2015)(unpublished data PROBE-C [4]). |
| **Proportion of MSM living with HIV infected with HCV upon HIV diagnosis** | Prior range: 0.35-0.95%, uniform  Posterior: 0.42% [95% I 0.35-0.53%] | HIV Seroconverter cohort unpublished – [5] |
| **Duration acute infection until spontaneous clearance** | Prior range: 3-9 months, uniform  Posterior: 6 months [95% I 3.5-8.5 months] | [2] |
| **SVR with IFN/RBV**  **<1 year from HCV infection**    **>1 year from HCV infection** | Prior range: 65-75%, uniform  Posterior: 67% [95% I 65-70%]  Prior range: 25-35%, uniform  Posterior: 30% [95% I 26-34%] | [6]  Weighted based on genotype distribution and SVR by genotype from a recent meta-analysis [7] |
| **Life expectancy from HIV diagnosis** | Varies over calendar time based on increasing ART coverage and earlier diagnosis (20-40 years from assumed HIV diagnosis and ART initiation at age 35 [Nakagawa et al, 2012; May et al., 2014])  Prior range:  **1996-99:** 18.82-21.18, uniform  **2000-02:** 27.02-29.38, uniform  **2003-05:** 30.62-32.58, uniform  **2006-08:** 34.62-36.58, uniform  Posterior:  **1996-99:** 20.1 [95% I 19.1-21.0]  **2000-02:** 28.1 [95% I 27.2-29.1]  **2003-05:** 31.6 [95% I 30.8-32.4]  **2006-08:** 35.6 [95% I 34.8-36.4] | Based on UK data as the proportion diagnosed on ART similar between UK and Germany (In 2004 70% in both UK and Germany [8], in 2019 98% in UK [9],and 87.6% in Germany in 2018 [10] and the median age at HIV diagnosis is similar (33 [IQR: 27-43; 11] vs 33 years in 2014) . We will fit a linear curve to life-expectancy data among diagnosed individuals who start ART at age 35 from 1997-2008 over calendar time among a UK cohort [12]. We will assume a continued increase in life expectancy from 2010 until a life expectancy of 73 years is reached (consistent with model estimates of life expectancy in a high diagnosis setting [13,14], and achieved in 2010 in the model) |
| **Excess liver-related mortality due for those with chronic HCV (annual)** | Prior range: 0.05-0.27, uniform]  Posterior: 0.14 [95% I 0.06-0.25] | [15]  [16] |
| **Background HCV incidence from outside population of MSM living with diagnosed HIV** | Prior range: 0-2/1,000 person-years, uniform  Posterior: 0.59/1,000person-years [95% I 0.18-1.03/1,000py] | Assumed similar to observed in the HIV-negative MSM population  [17] |
| **Proportion high risk** | Prior range: 0-30%, uniform  Posterior: 25% [95% I 19-29%] | Among a sub-sample of a large Internet survey among MSM in Europe in 2010, 5% of all MSM in Berlin reported consumption of drugs typically used at sex parties (ecstasy, amphetamines, crystal methamphetamine, mephedrone, GHB/GBL, ketamine, or cocaine) in the preceding 4 weeks, but MSM living with HIV were 5-times more likely to report this risk [18]. A German study among MSM living with HIV in 2014, reporting that 17% of MSM living with HIV report recent substance use [19]. |
|  |  |  |
| **Relative risk high risk compared to low risk** | Prior range: 0-100, uniform  Posterior: 25 [95% I 14-40] | Fitted mean value higher than relative risks of HCV infection among MSM living with HIV in Germany with associated individual behaviors, but range overlaps confidence intervals of data (such as frequent rectal trauma with bleeding adjusted odds ratio, AOR, 6.2 (95%CI 1.2-32.8), frequent receptive fisting without gloves AOR 5.7 (95%CI 1.5-21.7), and group sex AOR 3.5 (95%CI 0.8-14.5). Posterior relative risk similar to estimates related to HCV risk among MSM living with HIV in other settings for: sex with methamphetamine (AOR 28.6 [95%CI 1.84-443] 20) and reporting at least three sex risk factors [receptive unprotected anal intercourse [UAI], insertive UAI, receptive fisting, or insertive fisting] (AOR 23.5 (95%CI 9.5-58.3) [21]. |
| **Leaving rate from high risk (annual)** | Prior range: 0-0.5, uniform  Posterior: 0.04 [95% I 0-0.10] |  |
| **Initial HCV prevalence in 1996**  **Low risk**    **High risk** | Prior range: 0-1%, uniform  Posterior: 0.4% [95% I 0.08-0.9%]  Prior range: 0-1%, uniform  Posterior: 0.4% [95% I 0.05-0.8%] |  |
| **Infection rate** | Prior range: 0-0.2, uniform  Posterior: 0.06 [95% I 0.02-0.1] |  |
| **Number of MSM living with diagnosed HIV in 1996** | Prior range: 1,000-15,000, uniform  Posterior: 2,930 [95% I 1,110-6,185] |  |
| **Number of new entrants to MSM living with diagnosed HIV population each year** | Prior range: 3,000-4,000, uniform  Posterior: 3,540 [95% I 3,430-3,620] | Includes new HIV-diagnoses and those previously diagnosed and migrating to Berlin |

**References**

1. Thomson EC, Fleming VM, Main J, et al. Predicting spontaneous clearance of acute hepatitis C virus in a large cohort of HIV-1-infected men. Gut 2011; 60:83
2. Piroth L, Larsen C, Binquet C, et al. Treatment of acute hepatitis C in human immunodeficiency virus–infected patients: The HEPAIG study. Hepatology 2010; 52:1915-21.
3. Steininger K, Boyd A, Dupke S, et al. HIV-positive men who have sex with men are at high risk of development of significant liver fibrosis after an episode of acute hepatitis C. Journal of Viral Hepatitis 2017; 24:832-9.
4. Boesecke C, Nelson M, Ingiliz P, Lutz T. Does the Availability of New DAAs Influence Treatment Uptake in Acute Hepatitis C in HIV Coinfection? CROI Conference, Abstract 670 2015.
5. Jansen K, Thamm M, Bock C-T, et al. High Prevalence and High Incidence of Coinfection with Hepatitis B, Hepatitis C, and Syphilis and Low Rate of Effective Vaccination against Hepatitis B in HIV-Positive Men Who Have Sex with Men with Known Date of HIV Seroconversion in Germany. PLOS ONE 2015; 10:e0142515.
6. Boesecke C, Ingiliz P, Reiberger T, et al. Dual treatment of acute HCV infection in HIV co-infection: influence of HCV genotype upon treatment outcome. Infection 2016; 44:93-101
7. Davies A, Singh KP, Shubber Z, et al. Treatment Outcomes of Treatment-Naïve Hepatitis C Patients Co-Infected with HIV: A Systematic Review and Meta-Analysis of Observational Cohorts. PLoS ONE 2013; 8:e55373.
8. Yin Z, Brown A, Hughes G, Nardone A, Gill O, Delpech V. HIV in the United Kingdom: 2014 Report: data to end 2013. Public Health England, London, 2014.
9. Public Health England. Trends in HIV testing, new diagnoses and people receiving HIV-related care in the UK: data to end December 2019. Health Protection Report, Vol 14 No 20 https://assets.publishing.service.gov.uk/government/uploads/system/uploads/attachment_data/file/931964/hpr2020_hiv19.pdf
10. UNAIDS 2018. Germany Country Data. <https://www.unaids.org/sites/default/files/media_asset/unaids-data-2018_en.pdf>
11. Public Helath England. HIV in the United Kingdom: Towards Zero HIV transmissions by 2030 (2019 report – data to end 2018) https://assets.publishing.service.gov.uk/government/uploads/system/uploads/attachment_data/file/858559/HIV_in_the_UK_2019_towards_zero_HIV_transmissions_by_2030.pdf
12. May M, Gompels M, Delpech V, et al. Impact of late diagnosis and treatment on life expectancy in people with HIV-1: UK Collaborative HIV Cohort (UK CHIC) Study. BMJ 2011; 343:d6016.
13. Nakagawa F, Lodwick R, Smith C, et al. Projected life expectancy of people with HIV according to timing of diagnosis. AIDS 2012; 26:335-43.
14. May MT, Gompels M, Delpech V, et al. Impact on life expectancy of HIV-1 positive individuals of CD4+ cell count and viral load response to antiretroviral therapy. AIDS. 2014;28(8):1193-1202. doi:10.1097/QAD.0000000000000243
15. van der Helm J, Geskus R, Sabin C, et al. Effect of HCV Infection on Cause-Specific Mortality After HIV Seroconversion, Before and After 1997. Gastroenterology 2013; 144:751-60.e2.
16. Weber R, Sabin CA, Friis-Moller N, et al. Liver-related deaths in persons infected with the human immunodeficiency virus: the D:A:D study. Archives of internal medicine 2006; 166:1632-41.
17. Yaphe S, Bozinoff N, Kyle R, Shivkumar S, Pai NP, Klein M. Incidence of acute hepatitis C virus infection among men who have sex with men with and without HIV infection: a systematic review. Sex Transm Infect 2012; 88:558-64.
18. Schmidt AJ, Bourne A, Weatherburn P, Reid D, Marcus U, Hickson F. Illicit drug use among gay and bisexual men in 44 cities: Findings from the European MSM Internet Survey (EMIS). International Journal of Drug Policy 2016;38:4-12.
19. Esser S, Krotzek J, Dirks H, Scherbaum N, Schadendorf D. Sexual risk behavior, sexually transmitted infections, and HIV transmission risks in HIV-positive men who have sex with men (MSM) – approaches for medical prevention. JDDG: Journal der Deutschen Dermatologischen Gesellschaft 2017;15:421-428.
20. Fierer D, Factor S, Uriel A, Mullen M, Klepper A, van Seggelen W, et al. Sexual Transmission of Hepatitis C Virus Among HIV-infected Men Who Have Sex with Men - New York City 2005-2010. Morbidity and Mortaility Weekly Report 2011;60:945-950.
21. Danta M, Brown D, Bhagani S, Pybus OG, Sabin C, Nelson M, et al. Recent epidemic of acute hepatitis C virus in HIV-positive men who have sex with men linked to high-risk sexual behaviours. AIDS 2007;21:983-991.
