# Supplementary figures and images for: Establishing a framework towards monitoring HCV microelimination among men who have sex with men living with HIV in Germany: A modeling analysis
Source: PLoS One. 2022 May 12;17(5):e0267853. doi: 10.1371/journal.pone.0267853 (PMC9098082; doi:10.1371/journal.pone.0267853)

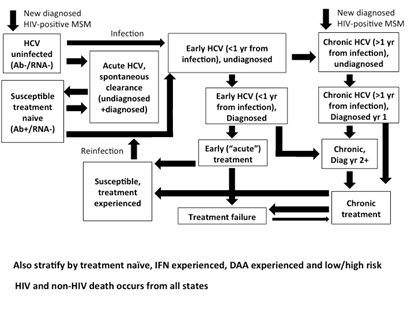

Supplement: S1 Fig — (TIF) [file pone.0267853.s002.tif]

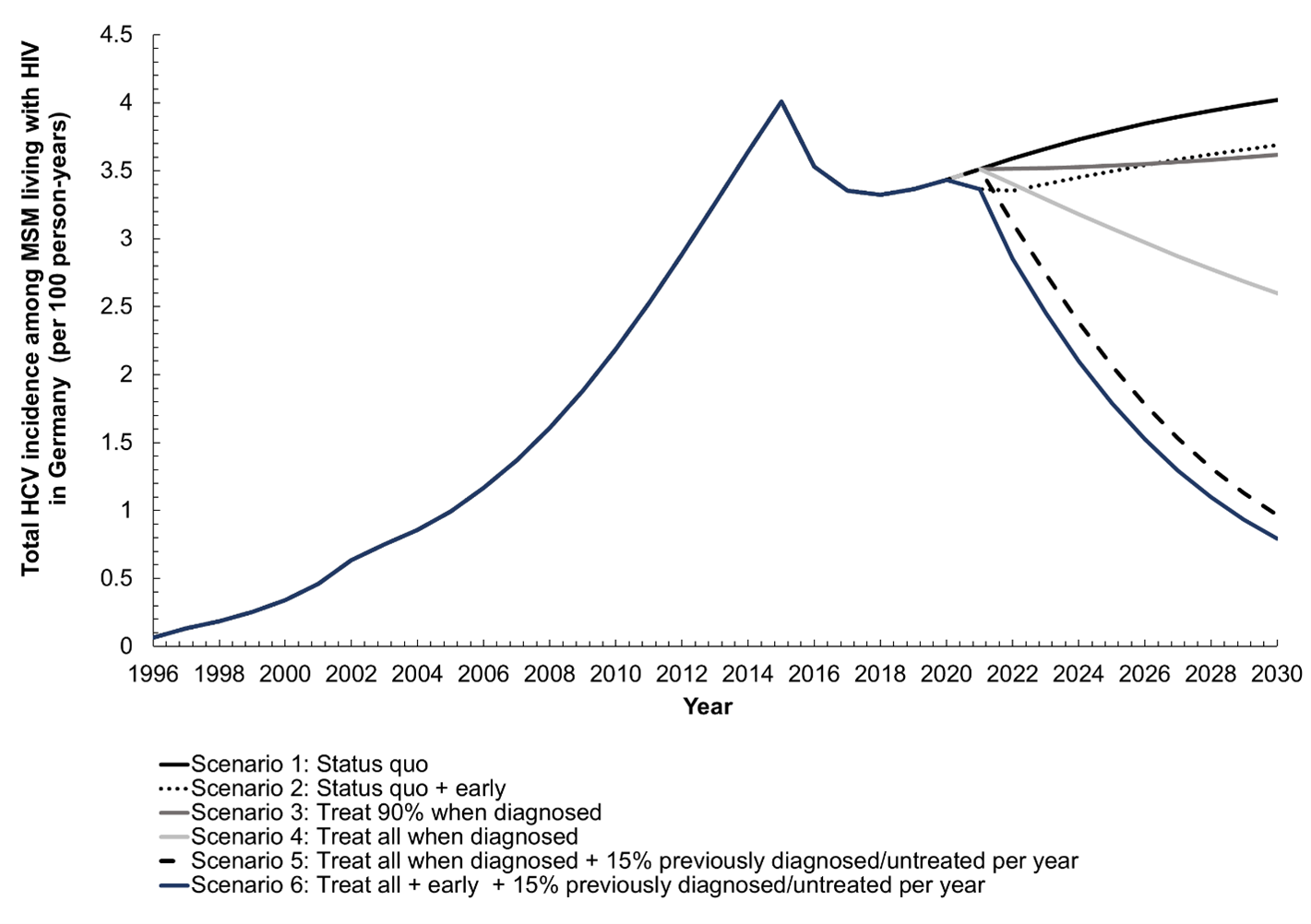

Supplement: S2 Fig — Primary incidence is defined as the incidence of first HCV infection. ‘Early’ refers to all newly diagnosed HCV infections treated within 3 months. (TIF) [file pone.0267853.s003.tif]

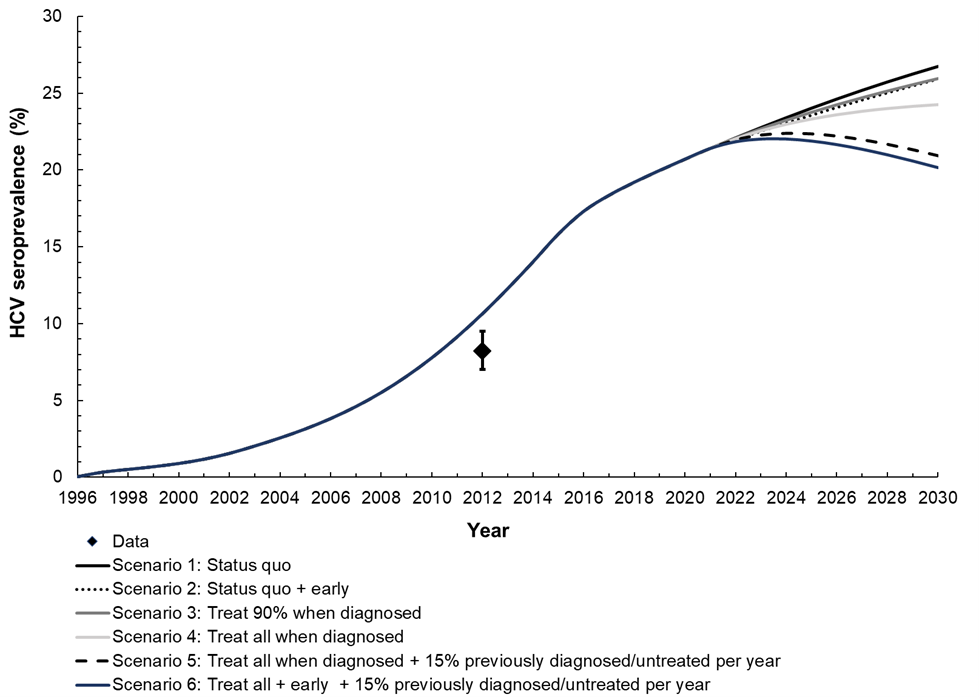

Supplement: S3 Fig — Data point is HCV seroprevalence estimate of 8.2% in 2012. ‘Early’ refers to all newly diagnosed HCV infections treated within 3 months. (TIF) [file pone.0267853.s004.tif]

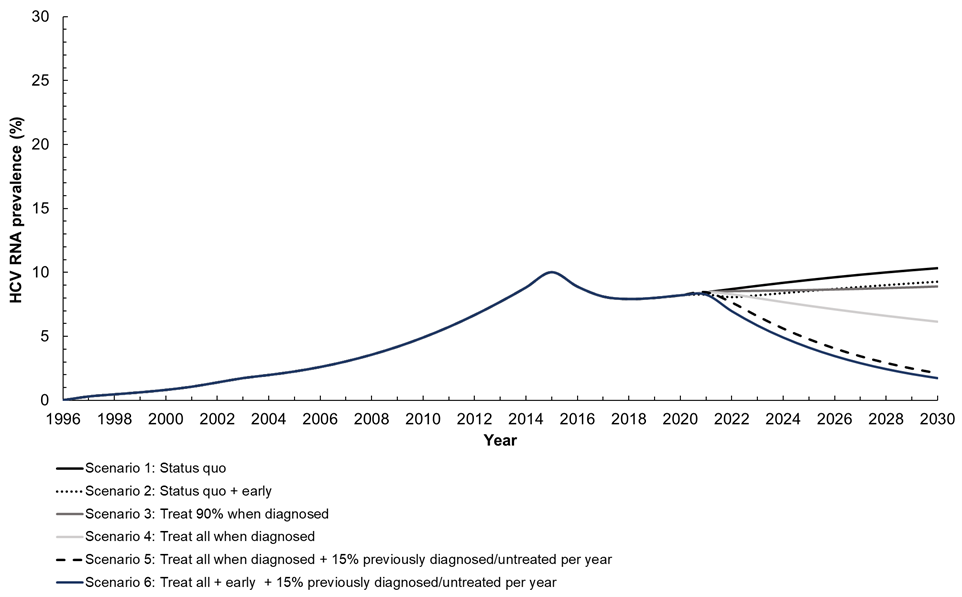

Supplement: S4 Fig — ‘Early’ refers to all newly diagnosed HCV infections treated within 3 months. (TIF) [file pone.0267853.s005.tif]

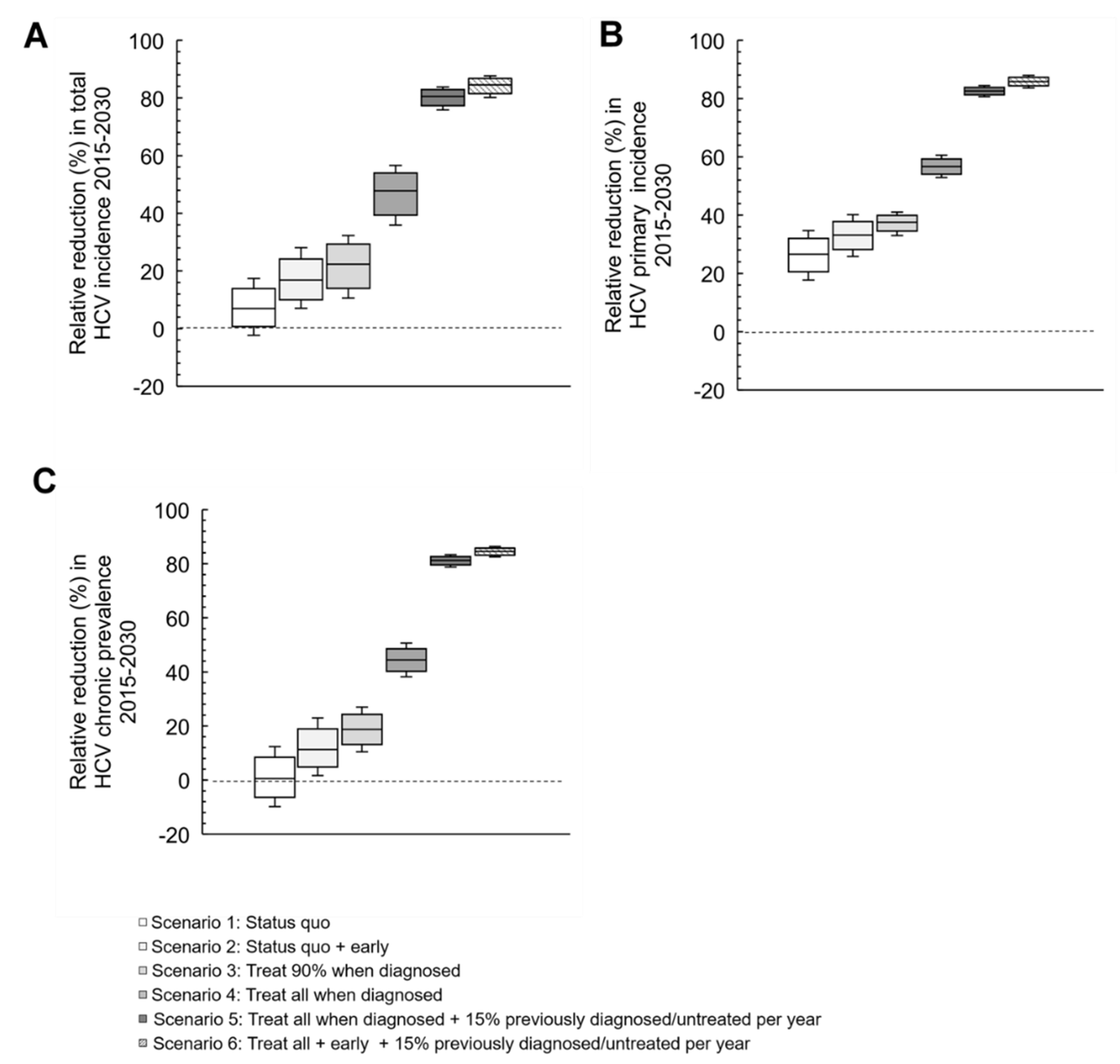

Supplement: S5 Fig — A). Relative reductions in total HCV incidence among MSM living with diagnosed HIV in Germany between 2015–2030. B). Relative reductions in primary HCV incidence among MSM living with diagnosed HIV in Germany between 2015–2030. C). Relative reductions in HCV chronic prevalence among MSM living with diagnosed HIV in Germany between 2015–2030. HCV chronic prevalence is defined as RNA+ only. HCV: hepatitis C; MSM: Men who have sex with men. Primary incidence is defined as the incidence of first HCV infection. ‘Early’ refers to all newly diagnosed HCV infections treated within 3 months. (TIF) [file pone.0267853.s006.tif]
