# Supplementary material for: Establishing a framework towards monitoring HCV microelimination among men who have sex with men living with HIV in Germany: A modeling analysis
Source: PLoS One. 2022 May 12;17(5):e0267853. doi: 10.1371/journal.pone.0267853 (PMC9098082; doi:10.1371/journal.pone.0267853)
Supplement: Attachment — Submitted filename: PONE-D-22-03984 reviewer comment.docx [file pone.0267853.s007.docx]

This manuscript described “Establishing a framework towards monitoring HCV microelimination among HIV-positive men who have sex with men in Germany: A modeling analysis.” The result presents that HCV elimination among HIV+ MSM in Germany requires further DAA scale-up among those newly diagnosed combined with efforts to treat those previously diagnosed but untreated.

In general, this is a well-written manuscript. Other points in this manuscript needed to be clarified are listed below:

1. HIV+MSM : I will suggest “MSM living with HIV” in aspect of terminology

Minor revisions:

1. Page 4, line 83: the subject is missing, Although “what” often initiated…
2. Page 13, line 202: please make sure the number of confidence interval is correct 2.5% and 97.5% ?
3. References: please unify the standard form of references, such number of authors, doi, PMID, etc….
